# Associations between neutrophil-percentage-to-albumin ratio level and all-cause mortality and cardiovascular disease-cause mortality in general population: evidence from NHANES 1999–2010

**DOI:** 10.3389/fcvm.2024.1393513

**Published:** 2024-09-25

**Authors:** Yuting Liu, Zifeng Qiu, Geng Shen, YangYang Sun, Jiarong Mei, Zhihao Liu, Leyi Wang, Jianping Li

**Affiliations:** ^1^ Department of Cardiology, Peking University First Hospital, Beijing, China; ^2^ Institute of Cardiovascular Disease, Peking University First Hospital, Beijing, China

**Keywords:** inflammation, neutrophil-percentage-to-albumin ratio, all-cause mortality, cardiovascular disease, national health and nutrition examination survey

## Abstract

**Introduction:**

Chronic inflammation is a recognized independent risk factor for cardiovascular disease (CVD), highlighting the need for reliable inflammatory indicator to predict CVDs. As an inflammatory indicator which has been proved to have predictive value for prognosis of CVDs, neutrophil percentage-to-albumin ratio (NPAR) has obtained increasing attention, but further research is needed to confirm the relationship with mortality in the general population.

**Method:**

This prospective cohort study included 21,317 individuals who participated in the National Health and Nutrition Examination Survey (NHANES) from 1999 to 2010, where baseline characteristics and NPAR level were extracted. Data for CVD and all-cause mortality were acquired by linking the cohort database with the National Death Index through December 31, 2019. We employed restricted cubic spline analyses to examine the nonlinear association. Weighted Kaplan–Meier curves with log-rank tests were conducted to access cumulative survival differences across different NPAR results. Multivariable Cox proportional hazards regression models were used to compute hazard ratios and 95% CIs. Receiver Operating Characteristic (ROC) curves were used to compare predictive value of NPAR with systemic immune inflammation index (SII) and neutrophils percent.

**Results:**

In this cohort study, during 270,014 person-years of follow-up, 4,074 all-cause deaths and 1,116 CVD-cause deaths were documented. NPAR levels exhibited significant nonlinear associations with both CVD-cause (*P* = 0.018 for nonlinearity) and all-cause mortality (*P* < 0.001 for nonlinearity). Participants in the highest NPAR tertile had a significantly increased risk of all-cause mortality (HR: 1.46, 95% CI: 1.33–1.61) and CVD-cause mortality (HR: 1.54, 95% CI: 1.32–1.80) compared to those in the lowest tertile in the fully adjusted model, while no association was detected for individuals in the middle tertile. Further ROC analysis confirmed that NPAR had higher predictive value than neutrophil percent segment and SII.

**Conclusions:**

Elevated NPAR level was significantly associated with an increased risk of all-cause and CVD-cause mortality in general population. The high predictive value of NPAR, combined with the easy-to-calculate property, suggests that its potential as a novel inflammatory indicator is worthy of further investigation.

## 1 Introduction

Cardiovascular disease (CVD) stands as the leading cause of mortality all over the world with an escalating prevalence. There have been 607.64 million recorded CVD cases globally, impacting 48.6% of world population in 2022 (1). As a major disease burden, CVD led to 19.05 million deaths in 2020, marking an 18.71% increase since 2010 and contributing to 12.5% of global mortality over the past decades (2, 3).

The association between cardiovascular pathogenesis and chronic inflammation is widely acknowledged (4). A growing number of novel inflammation indicators that have predictive and prognosis value on CVD have been discovered, such as neutrophil-to-lymphocyte ratio (NLR), platelet-to-lymphocyte ratio (PLR), and systemic immune inflammation index (SII) (5–8). The neutrophil percentage-to-albumin ratio (NPAR), calculated by dividing neutrophil percentage by albumin level, has garnered significant attention as a potential biomarker of systematic inflammation, concerning the essential roles played by these two physiological biomarkers. Neutrophils, as crucial innate immune cells in inflammatory response, are recognized to be associated with morbidity and mortality of CVD (9). Serum albumin, playing a role in substance transport and osmosis regulation, demonstrates potential protective effects against multiple CVDs, which attribute not only to its anti-inflammatory activity, but also to its antioxidant effects and antiplatelet aggregation properties (10–12).

Pervious hospital-based analyses have shown a significant association between NPAR and one-year mortality in patients with atrial fibrillation (13), coronary artery disease (14), myocardial infarction (15)and heart failure (16), highlighting its predictive value for prognosis of CVD. For patients with stroke, a high NPAR value was proved to be associated with stroke associated pneumonia (17) and other infections (18). Only limited studies reported association of NPAR with mortality in community-based population, including patients with chronic obstructive pulmonary disease (19) and non-alcoholic fatty liver diseases (20). Notably, a study focused on the association between NPAR and mortality in heart failure patients revealed a higher predictive value of NPAR compared to NLR or PLR, suggesting its potential value in prognosis (21). However, few study has focused on the predictive value of NPAR for CVD in the general population. Therefore, to assess the prognostic value of NPAR in primary prevention, this study aimed to evaluate the association between NPAR and CVD and all-cause mortality in general population.

## 2 Materials and methods

### 2.1 Study population

We utilized the National Health and Nutrition Examination Survey (NHANES), a nationally represented survey conducted by the National Center for Health Statistics of the Centers for Disease Control and Prevention which can be considered as a representative sample of the noninstitutionalized US civilian population. Data acquisition included information on health conditions, health related behaviors, and demographic and socioeconomic characteristics of participants obtained through standardized questionnaires administrated by skilled interviewers during study's recruitment phase. Physical measurements and laboratory tests of all participants were conducted by professional medical practitioner within mobile examination centers. A comprehensive explanation of NHANES sampling and analytic methodologies is accessible in a published guideline (22). This study adhered to the Strengthening the Reporting of Observational Studies in Epidemiology (STROBE) reporting guideline designed for cohort studies. The NHANES study protocols received approval from the Institutional Review Board of the National Center of Health Statistics, and all participants provided informed written consent upon enrollment. No compensation or any incentives were offered to participants in this study.

In our study, we examined participants from NHANES recruited between 1999 and 2010, all of whom were aged over 20 years old at study recruitment. Exclusion criteria were as follows: (1) Pregnancy; 2) Lack of mortality data or being lost to follow-up; (3) Failure to provide neutrophil percent or albumin level data; (4) Diagnosis of cancer at study recruitment or absence of cancer-related information.

### 2.2 Laboratory measurement and index calculation

In the NHANES study, blood samples were processed and frozen at −20°C, and subsequently tested by the National Center for Environmental Health. A comprehensive description of the laboratory methods can be access on the NHANES website. The NPAR was calculated by dividing the percentage of neutrophil by albumin level (unit as gram per deciliter), using the same blood sample following the formula: (Neutrophil percentage (%) * 100/Albumin (g/dl)). The SII was calculated as (P x N)/L, where P, N, and L represent the peripheral platelet, neutrophil, and lymphocyte counts respectively.

### 2.3 Ascertainment of mortality

Data for deaths were acquired by linking the cohort database with the National Death Index through December 31, 2019. All-cause mortality was defined as any cause of death. CVD mortality was specifically defined using the International Statistical Classification of Diseases and Related Health Problems, Tenth Revision codes I00 to I09, I11, I13, I20–I51, and I60–I69.

### 2.4 Assessment of covariates

In our study, race and ethnicity were self-reported during interviews and categorized as Mexican American, non-Hispanic Black, non-Hispanic White, and others (including other Hispanic, other non-Hispanic races and non-Hispanic multiracial). Body mass index (BMI) was computed by dividing weight in kilograms by the square of heights in meters, and categorized as lower than 28 kg/m^2^ or higher. Education levels were categorized as “under high school” [including less than a 9th-grade education or those in 9–11th-grade category (includes 12th grade with no diploma)], “high school” [including high school graduate, general educational development (GED) or equivalent], “college or more” (including some college or an AA degree, as well as those with a college graduate or above). The ratio of family income to the poverty threshold was calculated as the poverty income ratio (PIR). Marital status was categorized into married, widowed, divorced, separated, never married, living with a partner, refused, and do not know, as presented in the NHANES primary file. In our study, individuals in the married status or living with a partner were collectively classified as “married”. Individuals in the widowed, divorced or separated status were categorized as “unmarried”. Participants were further classified as never smokers (never smoked or smoked less than100 cigarettes in life and quit smoking), former smokers (smoked more than 100 cigarettes in life and quit smoking) and current smokers (smoked more than 100 cigarettes in life and is currently smoking).

Additionally, data on the physician-diagnosed history of diabetes, hypertension, and hypercholesterolemia were self-reported by participants. Medication information was collected by trained professionals who matched the participants-provided products with the drug database. In addition, levels of C-reactive protein (CRP), total cholesterol, high-density lipoprotein cholesterol, and indicators of liver function (levels of aspartate aminotransferase, alanine transaminase, gamma-glutamyl transpeptidase and lactate dehydrogenase) were measured at the time of recruitment.

We applied the Chronic Kidney Disease Epidemiology Collaboration (CKD-EPI) (23) to calculate estimated glomerular filtration rate (eGFR). The CKD-EPI Creatinine Equation (2021) is expressed as a single equation:

$$\mathrm{eGF}R_{cr}=142\times \min{\left(\frac{\mathrm{Scr}}{\kappa },1\right)}^{\alpha }\times \max\;{\left(\frac{\mathrm{Scr}}{\kappa },1\right)}^{-1.200}\times \;0.9938\times \mathrm{Age}\times 1.012\;[\mathrm{if}\;\;\mathrm{female}]$$

Where: Scr = standardized serum creatinine in mg/dl, κ is 0.7 (females) or 0.9 (males), α is −0.241 (female) or −0.302 (male), $\min\;\left(\frac{\mathrm{Scr}}{\kappa },1\right)$ is the minimum of $\frac{\mathrm{Scr}}{\kappa }$ or 1.0, $\max\;\left(\frac{\mathrm{Scr}}{\kappa },1\right)$ is the maximum of $\frac{\mathrm{Scr}}{\kappa }$ or 1.0, Age (years).

### 2.5 Statistical analysis

All analyses in the present study incorporated sample weights, clustering and stratification, considering the intricate sampling design of NHANES. The person-years calculation of every participant spanned from the date of recruitment to either the date of death or the end of follow-up (December 31, 2019), whichever occurred first. We conducted weighted Kaplan–Meier curves with log-rank tests to obtain cumulative survival differences across different NPAR results.

To explore the nonlinear relationship of NPAR with all-cause and CVD-cause mortality, a restricted cubic spline analysis with 4 knots (5th, 35th, 65th, and 95th percentiles) was applied, using the 25th percentile chosen as reference. The analysis was conducted within the values ranging from the first to the 95th percentile to minimize potential outlier influence. Nonlinearity was assessed by the likelihood ratio test. In addition, based on the results of restricted cubic spline analyses, we applied tertiles of NPAR to examine the relationship between NPAR and mortality, using the lowest tertile as reference. Multivariable Cox proportional hazards regression models were employed to compute hazard ratios (HRs) and 95% confidence interval (CI) to assess the associations between NPAR levels and CVD and all-cause mortality. Schoenfeld residuals were used to test the proportional hazards assumption, and no violation was observed.

Two multivariable models were constructed for comprehensive analysis. Model 1 was a crude model that only incorporated NPAR. In Model 2, various covariates were used for adjustment, which included age (continuous, years), sex (male or female), race and ethnicity (self-reported as Mexican American, non-Hispanic Black, non-Hispanic White or other), educational level (under high school, high school and college or more), poverty income ratio (PIR) and marital status (never married, married and unmarried). In Model 3, additional adjustments were made, which included smoking status (never, ever, or current), history of diabetes, hypertension or hypercholesterolemia (yes or no), diabetes medication use (with or without), hypertension medication use (with or without), lipid lowering medication use (with or without), BMI (continuous, kg/m^2^), systolic blood pressure (SBP, continuous, mmHg) and diastolic blood pressure (DBP, continuous, mmHg).

Variables with missing values were imputed using the multiple imputation approach based on “mi” R package.

Furthermore, stratified analyses were conducted by various factors, including age (<65 or ≥65 years), sex (male or female), race and ethnicity (self-reported Mexican American, non-Hispanic White, non-Hispanic Black or Other), education level (under high school, high school and college or more), smoking status (never, ever, or current), hypertension diagnosis (yes or no), diabetes diagnosis (yes or no), hypercholesterolemia diagnosis (yes or no), BMI (<28 or ≥28 kg/m^2^), systolic blood pressure (<140 or ≥140 mmHg) and diastolic blood pressure (<90 or ≥90 mmHg). The significance of interactions was assessed using the *P* values for the interaction terms between NPAR and the stratified factors.

In this study, a series of sensitivity analyses were conducted as follows: (1) The main analyses were repeated according to quintiles of NPAR. (2) Participants with a history of CVD were excluded from the main analyses. (3) To minimize the potential reverse causation bias, participants who died within 2 years of follow-up were excluded. (4) To explore the potential role of traditional inflammation, blood lipid levels, or liver and kidney indices on any observed associations, we further adjusted for C-reactive protein (CRP) levels, lipid profiles (including total cholesterol, high-density lipoprotein cholesterol), estimated glomerular filtration rate (an indicator of kidney function) and indicators of liver function (including plasma alanine transaminase, aspartate aminotransferase, lactate dehydrogenase and gamma-glutamyl transpeptidase).

To compare the predictive abilities of the NPAR, neutrophil percent and SII for all-cause and cardiovascular morality, we generated receiver operating characteristic curves (ROC) and calculated the area under curve (AUC).

All analyses were conducted using R software version 4.3.2. A two-sided *P* value < 0.05 was set as the threshold for statistical significance. Data were analyzed between October 1, 2023, and January 05, 2024.

## 3 Results

**Figure 1:**
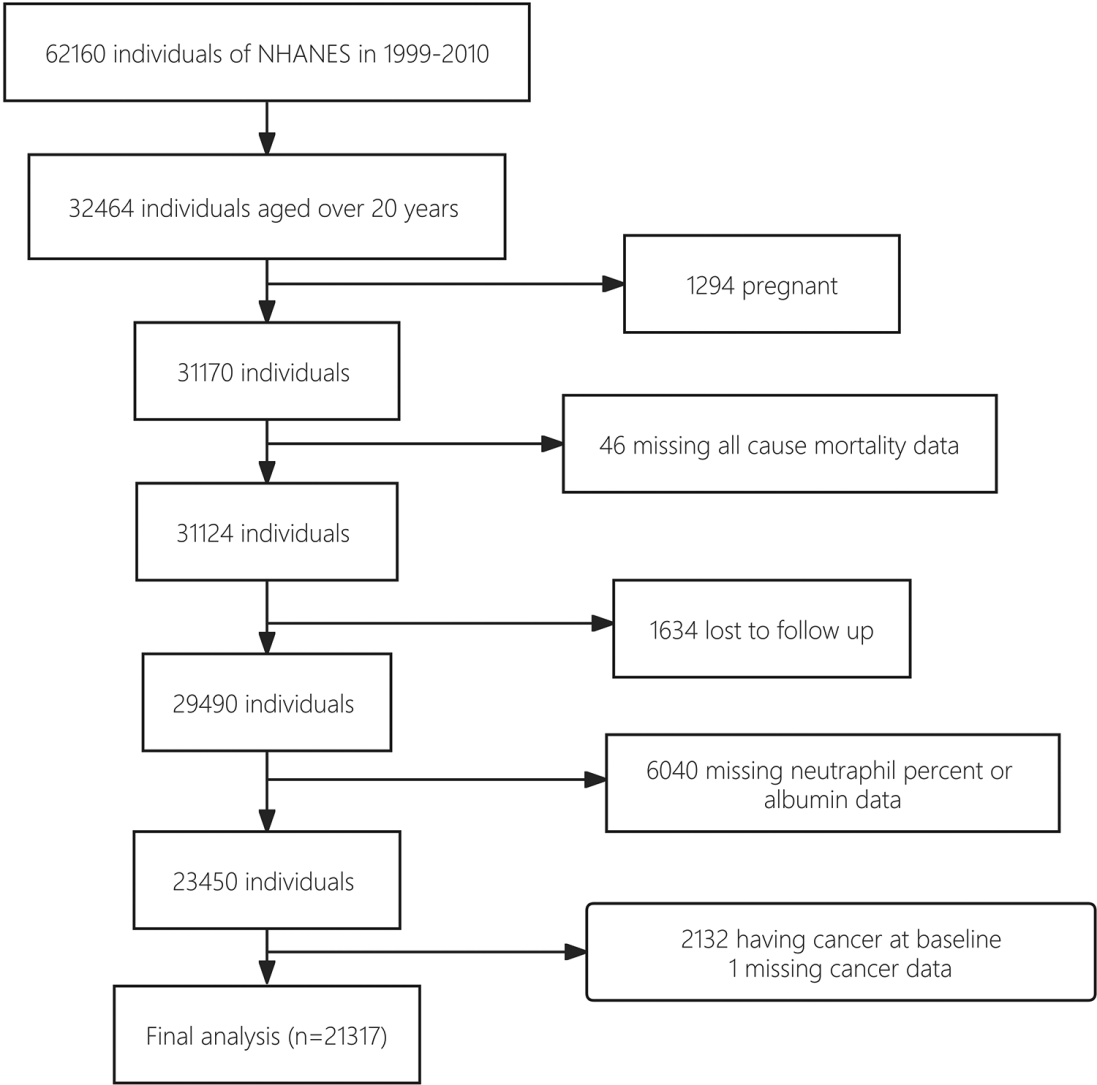
Flowchart.

The study cohort establishment is illustrated in a flowchart (Figure 1). A total of 62,160 NHANES participants who underwent interviews from 1999 to 2010 were identified. Of these, 32,464 subjects with a minimum age of 20 were initially chosen. Then, 21,317 subjects were included in the final cohort as the analytic samples after excluding 1,294 pregnant individuals, 46 with missing mortality data, 1,634 lost to follow up, 6,040 with missing data on neutrophil percentage or albumin levels, 3,132 having cancer at baseline, and 1 subject missing cancer data. This sample size represented a population of 148,855,370 US adults after weighting.

### 3.1 Baseline characteristics

Our cohort consisted of 21,317 participants (10,570 women [49.6%]; mean [SD] age, 48.9 [17.8] years). The baseline characteristics of participants divided by tertile of NPAR were demonstrated in Table 1. The ranges of NPAR for tertiles 1 through 3 were <12.59, 12.59 to 12.66, and ≥12.66. Compared with 7,101 participants in the lowest tertile of NPAR, 7,103 participants in the middle tertile were more likely to be older, women, non-Hispanic white, married, less educated, current smoked, having higher BMI, higher SBP, lower DBP, tending to have higher prevalence rates of hypertension, diabetes, hypercholesterolemia, and tending to use more antihypertensive, glucose lowering and lipid lowering drugs. Meanwhile, 7,107 participants in the highest tertile compared with 7,101 participants in the lowest tertile were likely to be older, women, non-Hispanic white, less educated, unmarried, current smokers, having higher BMI, higher SBP, lower DBP, tending to have higher prevalence rates of hypertension, diabetes and hypercholesterolemia, and tending to use more antihypertensive, glucose lowering and lipid lowering drugs.

**Table 1:** Baseline characteristic of study participants.

Characteristics	Total (*n* = 21,317)	Tertile 1 (*n* = 7,101)	Tertile 2 (*n* = 7,103)	Tertile 3 (*n* = 7,107)	*P*-value
		(<12.59)	(12.59–12.66)	(≥12.66)	
Sex, No. (%)					<0.001
Male	10,747 (49.9)	4,077 (58.8)	3,578 (50.1)	3,092 (40.3)	
Female	10,570 (50.1)	3,024 (41.2)	3,531 (49.9)	4,015 (59.7)	
Age, year	48.9 ± 17.8	46 ± 17.2	48.4 ± 17.5	52.3 ± 18.3	<0.001
Ethnicity, No. (%)					<0.001
Mexican American	4,531 (8.4)	1,418 (8.3)	1,656 (8.8)	1,457 (7.9)	
Non-Hispanic White	9,975 (69.6)	2,841 (64.4)	3,482 (71.6)	3,652 (72.7)	
Non-Hispanic Black	4,273 (11.1)	1,934 (15.1)	1,116 (8.4)	1,223 (9.9)	
Others	2,538 (10.9)	908 (12.1)	855 (11.2)	775 (9.5)	
Education level, No. (%)					0.004
<High school	6,621 (19.7)	2,187 (20.0)	2,118 (18.6)	2,316 (20.7)	
High school	5,071 (25.2)	1,598 (23.5)	1,752 (26.0)	1,721 (26.2)	
College or more	9,625 (55.1)	3,316 (56.5)	3,239 (55.4)	3,070 (53.2)	
Family income to poverty ratio	2.5 ± 1.6	2.6 ± 1.6	2.6 ± 1.6	2.4 ± 1.6	<0.001
Marital status, No. (%)					<0.001
Never married	3,709 (17.9)	1,442 (20.2)	1,155 (16.9)	1,112 (16.6)	
Married	12,903 (64.2)	4,348 (65.2)	4,472 (65.9)	4,083 (61.1)	
Unmarried	4,705 (17.9)	1,311 (14.5)	1,482 (17.1)	1,912 (22.3)	
Smoking status, No. (%)					0.011
Never	11,210 (52.4)	3,886 (53.7)	3,792 (53.0)	3,532 (50.3)	
Ever	5,211 (23.6)	1,640 (23.6)	1,736 (23.3)	1,835 (24.1)	
Current	4,896 (24.0)	1,575 (22.7)	1,581 (23.7)	1,740 (25.6)	
Self-reported disease					
Diabetes, No. (%)	2,270 (7.2)	565 (5.3)	688 (6.1)	1,017 (10.4)	<0.001
Hypertension, No. (%)	6,891 (27.6)	1,950 (23.3)	2,231 (27.2)	2,710 (32.7)	<0.001
High cholesterol	7,835 (35.0)	2,538 (33.2)	2,573 (35.1)	2,724 (36.9)	0.004
Antihypertensive drug, No. (%)	2,416 (18.4)	732 (14.7)	774 (17.2)	910 (23.4)	<0.001
Hypoglycemic drug, No. (%)	925 (6.2)	259 (4.3)	299 (5.4)	367 (9.0)	<0.001
Lipid.lowering drug, No. (%)	3,240 (13.1)	884 (10.9)	1,027 (12.1)	1,329 (16.4)	<0.001
BMI, kg/m^2^	27.8 (24.2, 32.1)	27 (23.8, 30.7)	27.8 (24.3, 31.9)	28.7 (24.8, 34)	<0.001
SBP, mmHg	124.5 ± 19.4	123.6 ± 18.4	123.9 ± 19.2	125.9 ± 20.6	<0.001
DBP, mmHg	70.3 ± 12.9	71.3 ± 12.2	70.5 ± 13	69.1 ± 13.5	<0.001

BMI, body mass index; SBP, systolic blood pressure; DBP, diastolic blood pressure.

### 3.2 Association between NPAR and CVD-cause and all-cause mortality

**Figure 2:**
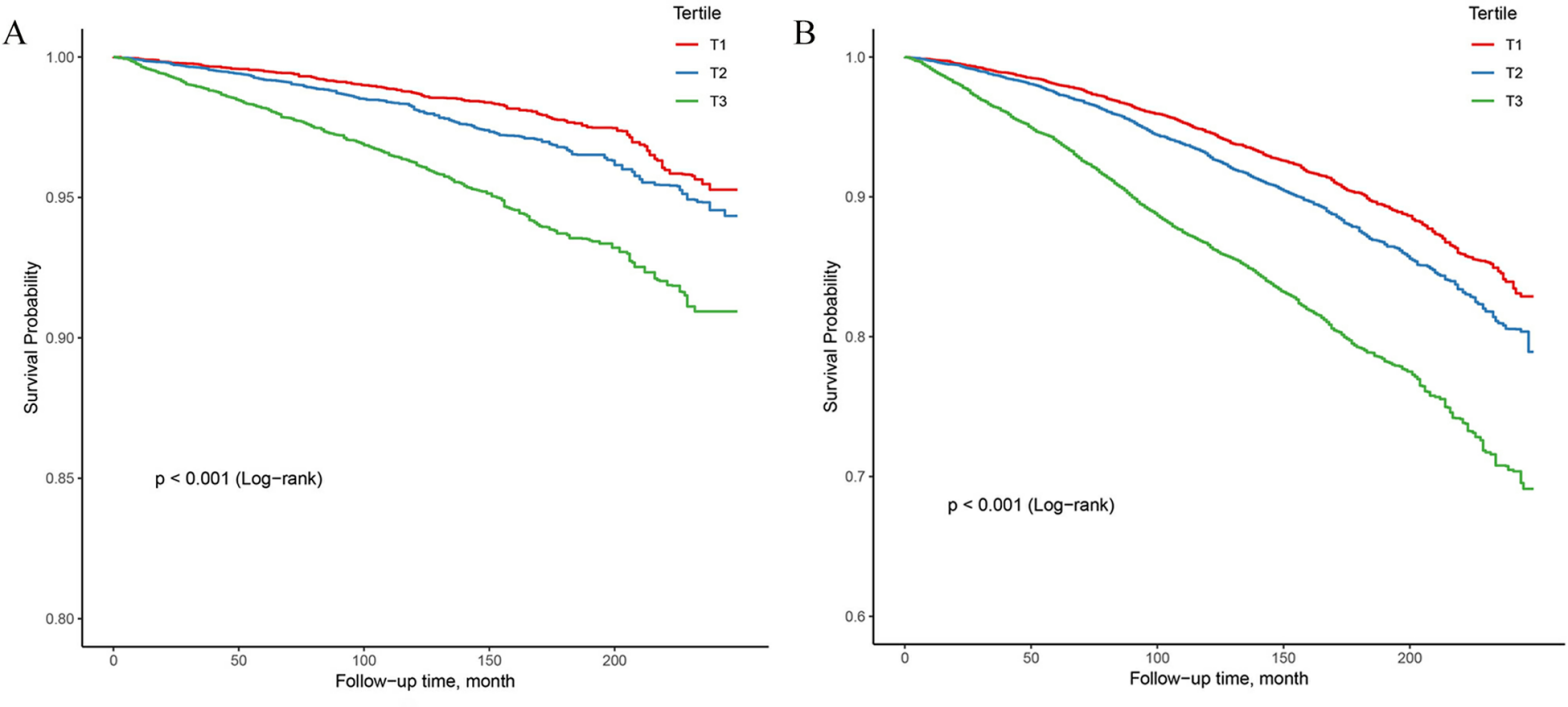
Kaplan–Meier survival analysis curves for all-cause and CVD-cause mortality. (**A**) Kaplan–Meier analysis for CVD-cause mortality; (**B**) Kaplan–Meier analysis for all-cause mortality.

In the study, 4,074 all-cause deaths and 1,116 CVD deaths were identified during 270,014 person-years of follow-up. Significant decrease in survival was observed with the gradual increase of NPAR level in both CVD and all-cause mortality analyses, as depicted in Kaplan-Miere curves in Figures 2A,B (*P* < .001). The HRs with 95% CI for multivariate models were presented in Table 2.

**Table 2:** Cox regression of the association between neutrophil percentage to albumin ratio and all-cause mortality and CVD-cause mortality among patients with hypertension.

Neutrophil percentage to albumin ratio	Person-y	No. of Events	Mortality Rate (per 1,000 Person-y)	Model 1^a^		Model 2^b^		Model 3^c^	
				HR (95% CI)	*P*-value	HR (95% CI)	*P*-value	HR (95% CI)	*P*-value
All-cause mortality									
Tertile 1 (<12.59)	94,682	962	10.16	Ref		Ref		Ref	
Tertile 2 (12.59–12.66)	91,949	1,203	13.08	1.27 (1.11, 1.44)	<0.001	1.11 (0.98, 1.25)	0.10	1.05 (0.94, 1.17)	0.42
Tertile 3 (≥12.66)	83,383	1,909	22.89	2.26 (2.04, 2.50)	<0.001	1.58 (1.44, 1.73)	<0.001	1.46 (1.33, 1.61)	<0.001
*P* for trend					<0.001		<0.001		<0.001
CVD-cause mortality									
Tertile 1 (<12.59)	94,682	251	2.65	Ref		Ref		Ref	
Tertile 2 (12.59–12.66)	91,949	325	3.53	1.36 (1.12, 1.66)	0.00	1.17 (0.97, 1.42)	0.10	1.09 (0.91, 1.32)	0.36
Tertile 3 (≥12.66)	83,383	540	6.48	2.62 (2.21, 3.10)	<0.001	1.72 (1.48, 2.00)	<0.001	1.54 (1.32, 1.80)	<0.001
*P* for trend					<0.001		<0.001		<0.001

CVD, cardiovascular disease; CI, confidence interval; HR, hazard ratio.

^a^ Model 1 was a crude model that only incorporates neutrophil percentage to albumin ratio.

^b^ Model 2 adjusted for age, sex, ethnicity, education level, family poverty income ratio and marital status.

^c^ Model 3 adjusted for age, sex, ethnicity, education level, family poverty income ratio, marital status, smoking status, self-reported hypertension, self-reported diabetes, self-reported high cholesterol, antihypertensive drug, hypoglycemic drug, lipid lowering drug, body mass index, systolic blood pressure, diastolic blood pressure.

**Figure 3:**
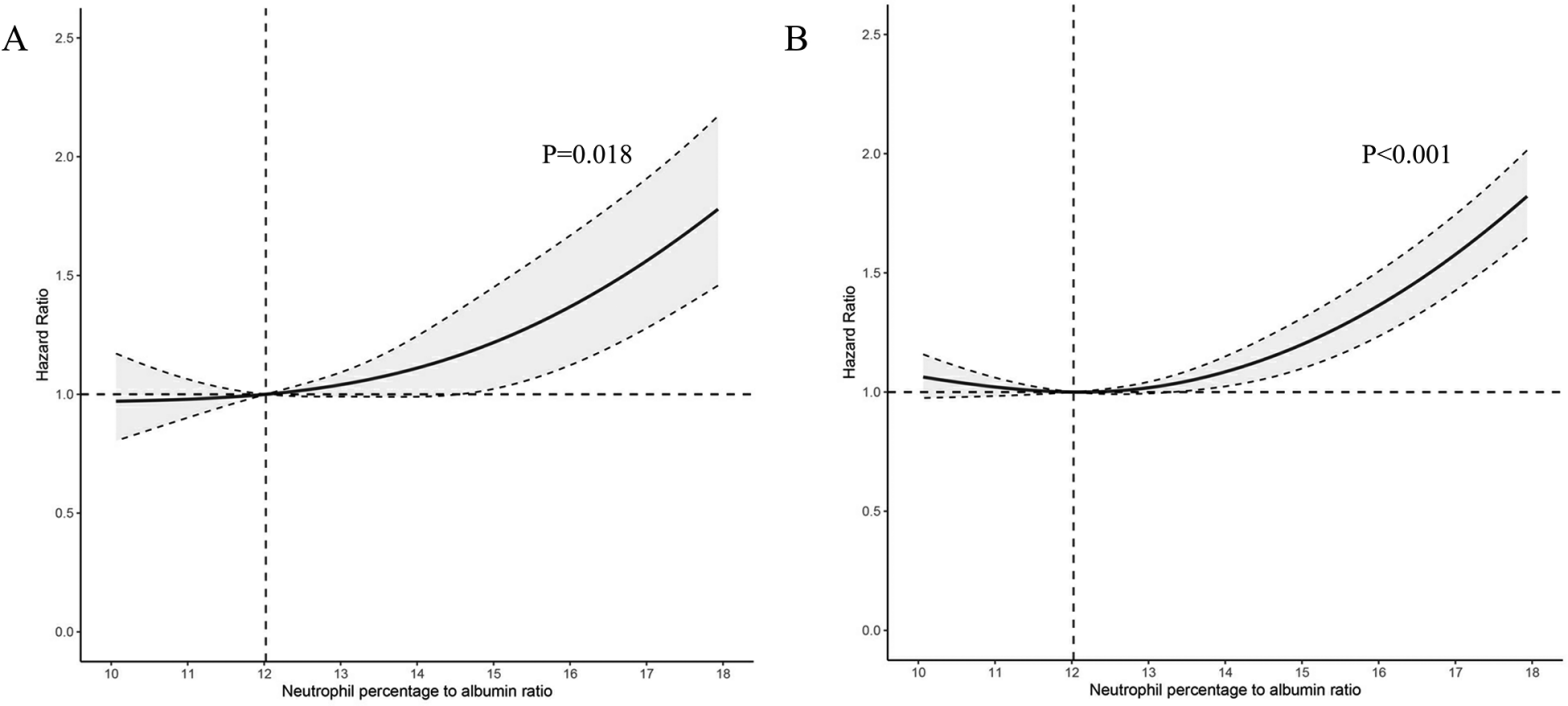
Association between NPAR level and all-cause and CVD-cause mortality. (**A**) CVD-cause mortality; (**B**) All-cause mortality; NPAR neutrophil percentage-to-albumin ratio; HR, Hazard Ratio.

A significant non-linear interaction between NPAR and CVD mortality was observed (*P* = 0.018 for no linearity) (Figure 3A). After multivariable adjustment, compared with the lowest tertile, participants had higher CVD mortality in the highest tertile of NPAR group (HR 1.54, 95% CI 1.32–1.1.80). Subgroup analyses revealed that NPAR had significant interaction with high cholesterol (*P* for interaction = 0.014) and BMI (*P* for interaction = 0.047) in prediction of CVD mortality. In most subgroups analyses revealed positive associations between the highest tertile and the lowest, except for Mexican American, education level lower than high school, current smokers, those with diabetes, and those without hypertension. Positive associations between the middle tertile and the lowest were only detected in participants aged 65 or older and those with BMI over 28 kg/m^2^.

Similar associations were observed between NPAR and all-cause mortality (*P* < .001 for no linearity) (Figure 3B). After adjusting for multi-variants, the HRs compared with the lowest tertile were 1.05 (95% CI 0.94–1.17) for the middle tertile and 1.46 (95% CI 1.33–1.61) for the highest tertile. All subgroup analyses showed similar results, with statistically significant associations detected between the highest and the lowest tertile. High cholesterol was the only factor we found to significantly interact with NPAR (*P* for interaction = 0.022). Additionally, in the subgroup of individuals with age over 65 years, the HR was 1.24 (95% CI 1.10–1.39) in the middle tertile compared with the lowest tertile. All subgroup analyses results were presented in Table 3.

**Table 3:** Subgroup analysis of the association of neutrophil percentage to albumin ratio with all-cause mortality and CVD-cause mortality.

CVD-cause mortality					All-cause mortality			
Subgroups	Hazard ratio (95% CIs) by tertile			*P* for interaction	Hazard ratio (95% CIs) by tertile			*P* for interaction
	Tertile 1	Tertile 2	Tertile 3		Tertile 1	Tertile 2	Tertile 3	
Sex				0.931				0.931
Male	Ref	1.20 (0.93, 1.56)	1.44 (1.08, 1.91)		Ref	1.06 (0.90, 1.25)	1.45 (1.24, 1.69)	
Female	Ref	0.97 (0.74, 1.27)	1.67 (1.37, 2.05)		Ref	1.03 (0.88, 1.21)	1.47 (1.30, 1.67)	
Age, year				0.076				0.076
<65	Ref	0.99 (0.69, 1.42)	1.74 (1.24, 2.44)		Ref	1.00 (0.80, 1.26)	1.59 (1.32, 1.91)	
≥65	Ref	1.26 (1.03, 1.54)	1.63 (1.36, 1.95)		Ref	1.24 (1.10, 1.39)	1.63 (1.46, 1.81)	
Ethnicity				0.786				0.076
Mexican American	Ref	1.03 (0.59, 1.80)	1.54 (0.90, 2.64)		Ref	0.95 (0.73, 1.23)	1.40 (1.12, 1.76)	
Non-Hispanic White	Ref	1.16 (0.92, 1.47)	1.55 (1.24, 1.94)		Ref	1.06 (0.93, 1.21)	1.38 (1.23, 1.55)	
Non-Hispanic Black	Ref	0.97 (0.65, 1.44)	1.80 (1.25, 2.57)		Ref	1.03 (0.79, 1.35)	1.75 (1.48, 2.07)	
Others	Ref	0.78 (0.28, 2.17)	1.22 (0.57, 2.58)		Ref	0.90 (0.48, 1.68)	1.92 (1.38, 2.67)	
Education level				0.095				0.244
<High school	Ref	1.21 (0.90, 1.62)	1.28 (0.95, 1.72)		Ref	1.03 (0.88, 1.21)	1.45 (1.24, 1.69)	
High school	Ref	0.99 (0.61, 1.61)	1.91 (1.22, 2.99)		Ref	1.11 (0.91, 1.35)	1.73 (1.44, 2.09)	
College or more	Ref	1.05 (0.68, 1.62)	1.58 (1.11, 2.26)		Ref	1.05 (0.86, 1.28)	1.32 (1.12, 1.55)	
Smoking status				0.869				0.417
Never	Ref	1.19 (0.87, 1.62)	1.65 (1.27, 2.14)		Ref	1.02 (0.86, 1.21)	1.52 (1.31, 1.77)	
Ever	Ref	1.08 (0.73, 1.60)	1.38 (1.00, 1.91)		Ref	1.08 (0.91, 1.28)	1.49 (1.26, 1.77)	
Current	Ref	1.00 (0.65, 1.53)	1.48 (0.96, 2.27)		Ref	1.07 (0.86, 1.34)	1.32 (1.06, 1.63)	
Diabetes				0.449				0.529
No	Ref	1.08 (0.85, 1.37)	1.60 (1.34, 1.91)		Ref	1.05 (0.92, 1.19)	1.43 (1.29, 1.58)	
Yes	Ref	1.15 (0.76, 1.72)	1.33 (0.82, 2.14)		Ref	1.04 (0.81, 1.34)	1.57 (1.27, 1.96)	
Hypertension				0.608				0.210
No	Ref	1.06 (0.76, 1.49)	1.30 (0.94, 1.79)		Ref	1.03 (0.86, 1.23)	1.30 (1.10, 1.53)	
Yes	Ref	1.15 (0.86, 1.53)	1.72 (1.39, 2.14)		Ref	1.09 (0.94, 1.26)	1.64 (1.44, 1.88)	
High cholesterol				0.014				0.022
No	Ref	0.94 (0.70, 1.27)	1.64 (1.25, 2.14)		Ref	0.96 (0.82, 1.12)	1.49 (1.31, 1.69)	
Yes	Ref	1.15 (0.99, 1.33)	1.43 (1.24, 1.65)		Ref	1.15 (0.99, 1.33)	1.43 (1.24, 1.65)	
BMI, kg/m^2^				0.047				0.460
<28	Ref	0.91 (0.71, 1.18)	1.49 (1.21, 1.84)		Ref	1.12 (0.99, 1.27)	1.48 (1.30, 1.69)	
≥28	Ref	1.32 (1.00, 1.74)	1.66 (1.28, 2.16)		Ref	0.96 (0.82, 1.14)	1.42 (1.24, 1.62)	
SBP, mmHg				0.975				0.429
<140	Ref	1.06 (0.82, 1.38)	1.51 (1.22, 1.86)		Ref	1.00 (0.85, 1.17)	1.36 (1.20, 1.55)	
≥140	Ref	1.15 (0.85, 1.55)	1.60 (1.22, 2.09)		Ref	1.15 (0.98, 1.36)	1.65 (1.41, 1.93)	
DBP, mmHg				0.865				0.850
<90	Ref	1.10 (0.90, 1.34)	1.53 (1.31, 1.79)		Ref	1.04 (0.93, 1.18)	1.45 (1.32, 1.59)	
≥90	Ref	1.23 (0.49, 3.06)	2.77 (1.37, 5.60)		Ref	1.16 (0.69, 1.96)	2.10 (1.27, 3.47)	

Model adjusted for age, sex, ethnicity, education level, family poverty income ratio, marital status, smoking status, self-reported hypertension, self-reported diabetes, self-reported high cholesterol, antihypertensive drug, hypoglycemic drug, lipid lowering drug, body mass index, systolic blood pressure, diastolic blood pressure. The strata variable was not included when stratifying by itself.

BMI, body mass index (calculated as weight in kilograms divided by height in meters squared); CVD, cardiovascular disease; CI, confidence interval; HR, hazard ratio; NHANES, National Health and Nutrition Examination Survey.

### 3.3 Comparison of NPAR with SII and neutrophils percentage

**Figure 4:**
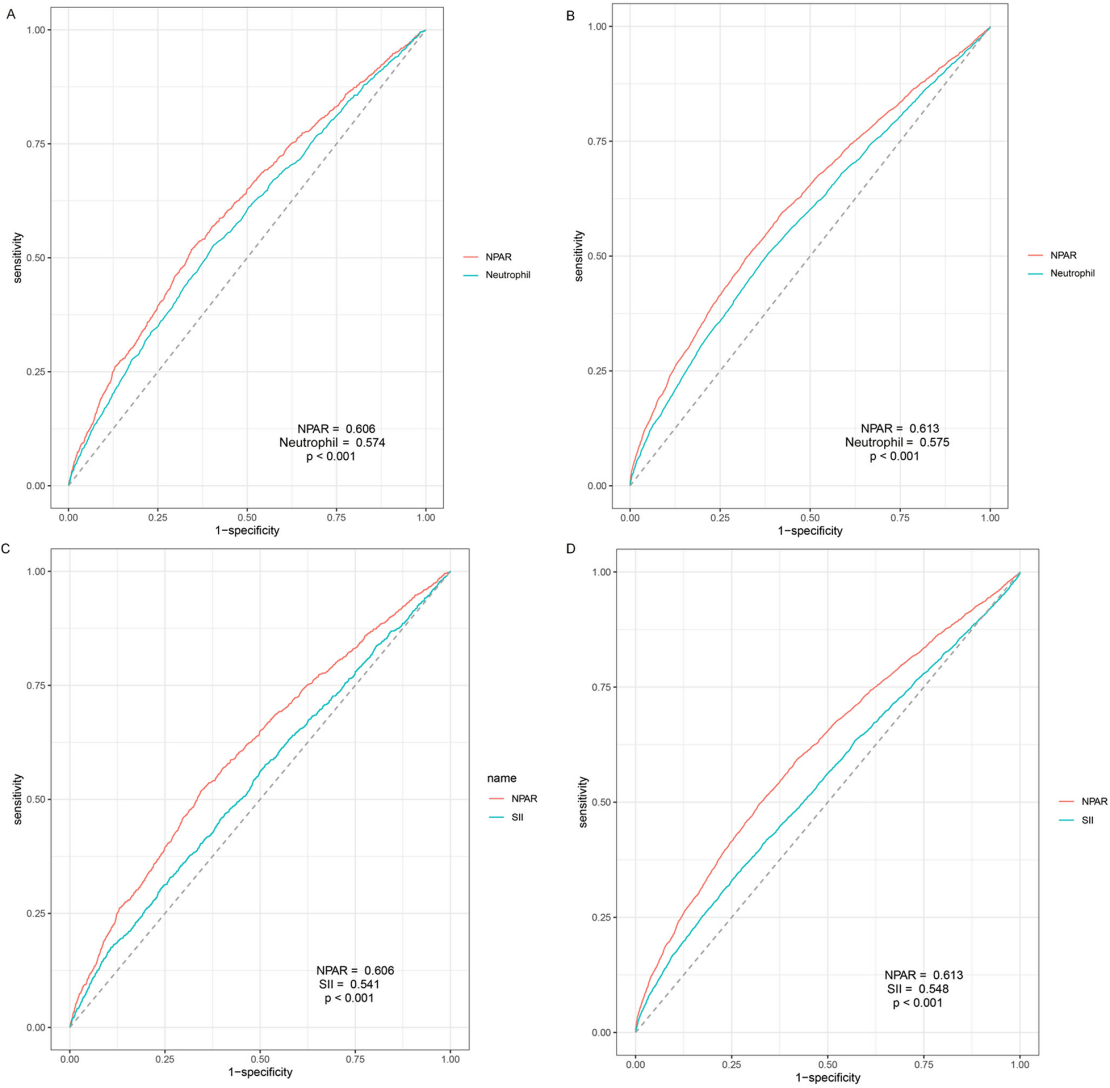
Time-dependent receiver-operating characteristic curves for NPAR and neutrophil and SII. (**A**) Prediction of CVD-cause mortality of NPAR and neutrophil percent; (**B**) Prediction of all-cause mortality of NPAR and neutrophil percent; (**C**) Prediction of CVD-cause mortality of NPAR and SII; (**D**) Prediction of all-cause mortality of NPAR and SII; NPAR, neutrophil percentage-to-albumin ratio; LBXNEPCT, Neutrophil percent; SII, systemic immune inflammation index.

The ROC analyses conducted to assess the predictive performance of NPAR, neutrophil percentage and SII in CVD-cause and all-cause mortality were presented in Figure 4. As expected, NPAR outperformed neutrophil percentage alone (0.606 vs. 0.574, *p* < .001) and showed a higher predictive value compared with SII (0.606 vs. 0.541, *p* < .001) in predicting CVD-cause mortality. Similar results were observed in predicting all-cause mortality (NPAR vs. Neu: 0.613 vs. 0.575, *p* < 0.001; NPAR vs. SII: 0.613 vs. 0.548, *p* < .001). The cut-off value of NPAR to predict all-cause mortality and CVD-cause mortality were 1,452.44 and 1,452.57 respectively. The sensitivity was 0.494 and 0.518, and the specificity was 0.680 and 0.656, respectively.

### 3.4 Sensitivity analysis

The results remain generally robust in sensitive analyses, except for individuals less than 2-year follow up (Supplementary Table S1), repeating the main analyses by quintiles of NPAR (Supplementary Table S2), or further excluding participants who had a history of CVD at baseline (Supplementary Table S3). Associations did not materially change with additional adjustments by CRP levels, lipid levels, liver function-related or kidney function-related indicators (Model 2, 3, 4, and 5 in Supplementary Table S4).

## 4 Discussion

In this large, prospective cohort study of US adults, we discovered significant non-linear associations between NPAR and both CVD-cause mortality and all-cause mortality in general population. The highest tertile of NPAR (>12.66) was statistically significant associated with high risk of CVD-cause and all-cause mortality compared to the lowest tertile (<12.59), while the middle level of NPAR (from 12.59–12.66) showed no significant association. Our findings were robust via a variety of stratified and sensitivity analyses, indicating that certain NPAR level can be used as a primary predictor of CVD-cause and all-cause mortality in individuals of different ages, sexes and ethnicities. The ROC analyses further demonstrated the superiority of NPAR over the neutrophil percentile and SII for predicting these outcomes.

In our analysis, the predictive value of NPAR on CVD-cause and all-cause mortality in general population is reported for the first time to our knowledge. NPAR, first introduced by Hehe Cui in 2019, demonstrated high predictive value for in-hospital mortality in patients with ST-elevated myocardial infarction (24). Subsequently, it has been widely used as an inflammation predictor in hospitalized patient, successfully predicting one-year mortality in patients with atrial fibrillation (13), coronary artery disease (14), myocardial infarction (15) and heart failure (16). Recently, there has been growing interest in using NPAR to predict mortality in community-based populations, including patients with chronic obstructive pulmonary disease (19) and non-alcoholic fatty liver diseases (20), but the association in general population remains unknown. While other inflammation biomarkers, such as monocyte-to-lymphocyte ratio (25), SII, System Inflammation Response Index (SIRI) (6), NLR (26), Hemoglobin, albumin, lymphocyte, and platelet (HALP) score (27), have shown predictive value on CVD-cause and all-cause mortality in general population, the predictive value of NPAR in the general population is needed to be examined.

The predictive value of NPAR may be attributed to the combined effects of neutrophil and albumin. Neutrophil, playing a crucial role in innate inflammation, has been proved to be deeply associated with multiple patterns of CVDs including ischemic heart disease, coronary artery disease, myocardial infarction, heart failure, and peripheral arterial disease in cohort studies based on large datasets (9, 28–30). Multiple studies also demonstrated the association between neutrophil level and both fatal and non-fatal CVDs (30, 31). The potential pathogenetic mechanism may be the inflammatory effects of neutrophils, including releasing excessive reactive oxygen species (ROS) (32), degranulation leading to the release of pro-inflammatory alarmins (such as S100A8/A9) (33) and proteases (such as myeloperoxidase) (34), and the formation of neutrophil extracellular traps (NETs) (35). These pro-inflammatory responses can induce severe oxidative stress and endothelium dysfunction, resulting in atherosclerosis and destabilization of existed plaque, and increasing potential risk of CVD (36).

Notably, in the ROC analysis, NPAR showed a higher predictive value for both CVD-cause and all-cause mortality compared to neutrophil percentile alone, indicating a superior value of combining albumin level. It has been previously reported that hypoalbuminemia independently predicted a higher risk of ischemic heart disease, heart failure, atrial fibrillation, stroke and venous thromboembolism (10). The potential impact of low albumin level on CVD may be related to impaired anti-inflammatory, antioxidant, and antithrombotic properties (37).

Subgroup analyses revealed a stronger association between NPAR and CVD-cause and all-cause mortality in individuals aged over 65. The potential mechanism may underlie the crosstalk between inflammation and ageing called “inflamm-aging” (38). This chronic, systemic inflammation developing with age has been proved to be related to decline in cardiovascular function through senescence-associated secretory phenotypes, including immunosenescence, epigenetic change and metabolism disorder, making elders more vulnerable to inflammation (39). We found significant differences between individuals with and without hypercholesterolemia in both CVD-cause and all-cause mortality, which may be attribute to the widely acknowledged interplay between cholesterol and chronic inflammation. Cholesterol accumulation triggers inflammatory responses including enhance of Toll-like receptor signaling, inflammasome activation, and the excessive production of monocytes and neutrophils (40). Both the accumulation of cholesterol and innate inflammatory response play crucial role in atherosclerosis, ultimately leading to CVDs (41). Additionally, significant differences were detected between individuals with BMI over 28 kg/m^2^ and less than 28 kg/m^2^ in the association of NPAR and CVD mortality, but not all-cause mortality. The adverse effects caused by higher BMI may be explained by obesity-related inflammatory status. Chronic inflammation has been considered as a major bridge between obesity and CVD (42), which may attribute to adipose tissue macrophages accumulation, excessive ROS releasing and endoplasmic reticulum (ER) stress (43).

In addition to neutrophil percentage, the predictive value of NPAR was proved to be superior to SII, a widely discussed novel inflammatory indicator associated with CVD and all-cause mortality (6). Given the advantages of easier calculation and measurement for NPAR, its potential will hold great significance.

## Strength and limitation

To the best of our knowledge, our study is the first to report the predictive value of NPAR in the general population, revealing significant robust associations between elevated NPAR and CVD and all-cause mortality. The analysis was conducted by restricted cubic spline method and further verified by multiple stratified and sensitivity analysis, which provided reliable results. As an easy-to-calculate and cost-effective inflammatory indicator, NPAR showed a higher predictive value for mortality compared to SII, indicating its promising utility in inflammatory status and cardiovascular prevention that needs further investigation. Moreover, our study benefited from using a national database which represented the US general population and had a sufficient length of follow-up period, suggesting that our results were representative.

Despite these strengths, there are some limitations in this study. Firstly, as an observational study based on a single dataset, our results need further confirmation from multicenter perspective cohort studies. Secondly, potential bias from NHANES should be discussed with caution. Recall bias might exist considering many sections of NHANES data were derived from interviews and questionnaires. The covariates were assessed at baseline and might change during follow-up. In addition, since NHANES only represents the US general population, caution is advised when we interpret results on other racial and demographic groups. Lastly, levels of neutrophil and albumin were based on a single measurement, which cannot reflect the dynamic changes of NPAR index.

## 5 Conclusion

Our study firstly unveils the significant association between elevated NPAR level and CVD-cause and all-cause mortality in general population, especially in individuals aged over 65 or with obesity. The robust prognostic value of NPAR was further verified by stratified and sensitivity analysis, and its superiority over SII and neutrophil percentage was demonstrated in ROC analyses. Given its easy-to-calculate nature and relatively higher predictive value, NPAR index with implications in public health, deserves greater attention in inflammatory assessment and cardiovascular prevention.

## Data Availability

The original contributions presented in the study are included in the article/Supplementary Material, further inquiries can be directed to the corresponding author.
